# Electrically driven heartbeat effect of gallium-based liquid metal on a ratchet

**DOI:** 10.3389/fbioe.2022.1094482

**Published:** 2023-01-12

**Authors:** Shutong Wang, Yue Zhang, Jiuyang Wang, Dongmei Ren, Zhenwei Yu

**Affiliations:** ^1^ Shenzhen Institute of Advanced Electronic Materials, Shenzhen Institutes of Advanced Technology, Chinese Academy of Sciences, Shenzhen, China; ^2^ College of Chemistry and Materials Engineering, Bohai University, Jinzhou, China; ^3^ Key Laboratory of Bio-inspired Materials and Interfacial Science, Technical Institute of Physics and Chemistry, Chinese Academy of Sciences, Beijing, China; ^4^ Key Laboratory of Bio-inspired Smart Interfacial Science and Technology of Ministry of Education, School of Chemistry, Beihang University, Beijing, China

**Keywords:** liquid metal, heartbeat, asymmetric structure, surface tension, droplet manipulation

## Abstract

The realization of the liquid metal heartbeat effect shows better controllability under non-periodic stimuli than spontaneous oscillation or periodic stimuli. However, adjusting the liquid metal heartbeat performance, drop spreading area, and frequency, solely by the magnitude of the voltage, has great limitations. Here, we demonstrate that the eGaIn drop can beat inside graphite ring electrodes under DC voltage in alkaline solutions on ratchet substrates. These sawtooth structures provide asymmetric textures which influence liquid metal deformation during the beating of the heart. We achieved heartbeat frequencies from 2.7 to 4.8 Hz, a 100% increase in the tunable frequency range compared to that on a flat surface. The oxidative spreading of the eGaIn drop on the ratchet substrate shows that the drop penetrates into the grooves of the sawtooth structure. Moreover, we investigated the physical mechanisms affecting the eGaIn heartbeat frequency and the influence on the spreading area of the eGaIn drop at various sawtooth sizes and orientations. These findings not only enhance our understanding of droplet manipulation on sawtooth-structured surfaces but also facilitate the design of microfluidic pump systems.

## 1 Introduction

Heart beating transports blood to all parts of the body, which is essential for mammals to maintain their body functions. The liquid metal heartbeat phenomenon shows a periodic oscillation similar to the beating of the heart. The well-known mercury heartbeat was discovered by Lippmann in 1873 (Lippmann 1873). Mercury changed its interfacial tension periodically in an acidic solution to achieve oscillation through close contact with an iron nail. Recently, this rhythmic oscillation has attracted attention as the non-toxic room-temperature liquid metal gallium and its alloy can replace mercury (Haynes et al., 2012; Tang et al., 2014; Hu et al., 2017; Yu et al., 2017) and offer potential applications on fluid pumps, timers, actuators, and logical stochastic resonance systems (Kumar et al., 2019; Wang et al., 2019; Ren et al., 2020; Liu et al., 2022).

Generally, liquid metal heartbeat phenomena or oscillations occur spontaneously (Yi et al., 2019; Chen et al., 2019; Wang et al., 2016) or by periodic stimulation, such as AC voltage (Verma et al., 2013; Yang et al., 2016; Jin et al., 2019) and noise (Kumar, Cruz&Parmananda 2019). Yi, Wang&Liu (2019) and Chen, Yang, Wang, Wang&Liu (2019) reported the self-powered oscillation of the aluminum-assisted liquid metal droplet due to the formation of a galvanic cell by the addition of aluminum. Verma, Contractor&Parmananda (2013) and Li et al. (2019) manipulated the oscillations of Hg and Galinstan, respectively, by electrochemically tuning the interfacial tension in AC voltage. However, liquid metal oscillations induced by spontaneous electrochemical reactions and programmable electric fields are difficult to tune the performance and need a synchronized process.

Compared with the aforementioned liquid metal heartbeats, aperiodic stimuli can better manipulate liquid metal beating. The DC voltage-triggered gallium drop heartbeat in the graphite ring electrode is one of the liquid metal phenomena we discovered (Yu et al., 2018a; Yun et al., 2020). Ge et al. (2022) presented an eGaIn heartbeat on the floating electrode in DC voltage. On the basis of using DC voltage, there have been various attempts to control the liquid metal heartbeat or oscillation such as substrate materials (Liu et al., 2022; Xue, Ge et al., 2022; Hu et al., 2016), shape (Li et al., 2022; Wang, Bingxing et al., 2022), and position (Ge, Tao, Liu, Song, Xue, Jiang&Ren 2022) of electrodes.

Learning from nature, shorebirds relied on asymmetric structures of the beak to transport water droplets into their mouths (Prakash et al., 2008; Wen et al., 2015; Liu et al., 2017; Xu et al., 2019; Wang, Fang et al., 2022). The asymmetric structure (Ju et al., 2012; Lagubeau et al., 2011) and wettability gradient on the surfaces play important roles in droplet manipulation. However, as the gallium-based liquid metal has large surface tension (>500 mN/m) (Liu et al., 2012; Yu et al., 2018b; Handschuh-Wang et al., 2021a; Handschuh-Wang et al., 2021b), the existing non-reactive wettability gradient surfaces have less influence with it. The asymmetric structure of the surface is an ideal choice to manipulate the liquid metal drop during the beating of the heart. In this work, we introduced a non-conductive substrate with a sawtooth structure for eGaIn heart beating. The heartbeat system consists of an eGaIn drop, sodium hydroxide, a graphite ring electrode, and a ratchet substrate. The heartbeat behavior was explored by tuning the orientation of the ratchet substrate, the size of the eGaIn drop, and the sawtooth structure. The Fourier transform was used to distinguish the periodic and aperiodic beating of eGaIn drops. In addition, the effect of the spread area and the frequency of the eGaIn heartbeat on the ratchet substrates were also systematically investigated. Liquid metal-spreading experiments reveal the beating mechanism on the ratchet substrate. Adjusting the size and orientation of the sawtooth structure enables the eGaIn heartbeat to obtain a wide range of frequencies, which provides insight for the application of microfluid pumps, micro-actuators, switches, and artificial muscle pumps (Zhang et al., 2016; Wang, X. et al., 2022).

## 2 Materials and methods

### 2.1 Materials

NaOH (500 g, AR, Xilong Science Co.), eGaIn (99.99%, Ga75In25, Wuhan Abestars International Trade Co., LTD.), a graphite ring electrode (1.0 mm), 3D printing prism plate with an isosceles right-angle ratchet shape and black photosensitive resin PEI (ratchet size 0.5, 1.0, 1.5, 2.0, and 2.5 mm, Supplementary Figure S1), 3D printer (UnionTech, D600), deionized water (water purifier, WP-R0-20B), microsampler (0.5 mL, Hamilton Tianjin Industrial Technology Co., LTD.), mini digital Protractor, programmable power supply (0∼30 V), 3D laser confocal microscope (Keyence, VK-X1100), iPhone (1080p, 60 fps), and a Sony Alpha 6000 camera with the FE 90 mm F2.8 Macro G OSS lens.

### 2.2 Experiment setup

An adjustable angle adjustment platform was placed on the horizontal table, and the angle of the angle adjustment platform was set to 0.8°, measuring with an inclinometer; the glass Petri dish was placed horizontally in the center of the platform, and when the graphite ring electrode was placed on the ratchet substrates, the whole setup was placed in the middle of the Petri dish; then, the eGaIn droplet was pipetted with a microsampler, and the eGaIn droplet was placed on the lower side in the graphite ring; the NaOH solution was poured into the Petri dish until the solution completely covered the surface of the liquid metal droplet. Finally, the positive electrode was connected to the graphite ring, and the negative electrode was placed in the electrolyte solution; the DC power supply voltage was adjusted to 8 V to start the experiment.

The melting point of room temperature liquid metal applied to mimic the heart was 15°C of eGaIn, the concentration of NaOH solution was fixed at 1.5 mol/L, and the voltage was 8 V. The droplets of eGaIn were discussed by adjusting their volumes (60, 80, 100, and 120 μL) on four different sizes of ratchet substrates with three different moving directions (base direction, leg direction, and channel direction) The liquid metal exhibited a periodic heartbeat on the prismatic plate, and the key to the periodic heartbeat was the design of a circular graphite ring electrode structure, and the dynamic process of droplet beating on the prismatic plate was recorded with a video camera.

### 2.3 Characterization

The advanced screenshot process of the liquid metal heartbeat video was performed by GOM Player software with 0.01 s interval. All the images were cropped at the size of the graphite ring by ImageJ software. The dynamic behavior of the eGaIn drop was captured using the TrackMate function in plugins. The obtained data were Fourier transformed, and the frequency of the eGaIn heartbeat was achieved. The deformation area of the eGaIn drop during the beating process was measured manually by ImageJ as well. The structural features of the graphite ring electrode and the ratchet substrates were photographed using a macro camera. Laser scanning imaging of the surface of the prismatic plate was performed using a 3D laser confocal microscope with x5 lens.

## 3 Results and discussion

### 3.1 Heart beating effect of the eGaIn drop on ratchet substrates

**FIGURE 1:**
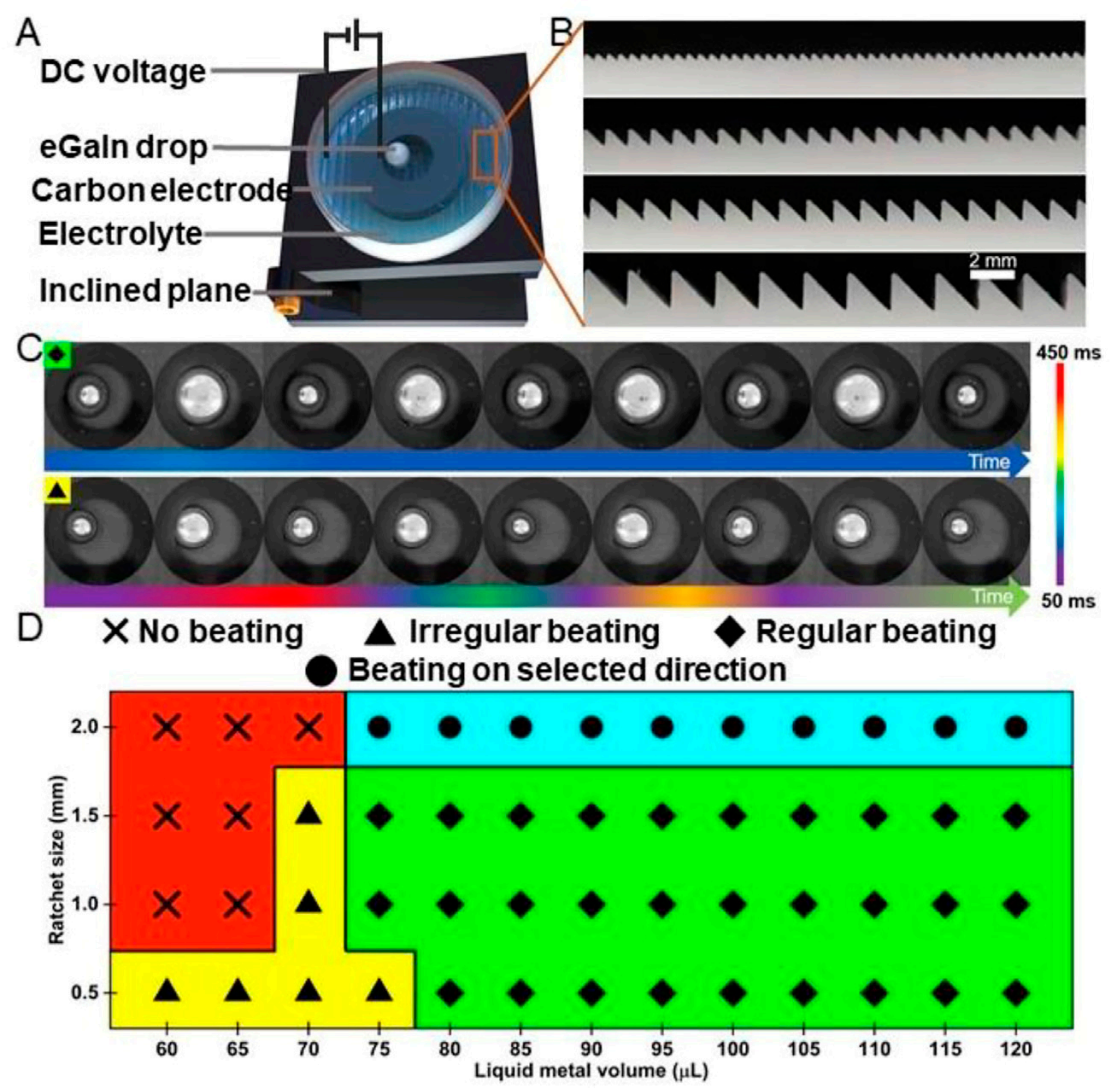
Heart beating effect of the eGaIn drop on ratchet substrates. **(A)** Schematic illustration of the experiment device. **(B)** Cross-section images of ratchet substrates with an isosceles right-angle shape (the sawtooth sizes are 0.5, 1.0, 1.5, and 2.0 mm). **(C)** Diagrammatic representation of four cycles of 120 and 60 μL eGaIn drops pounding in the channel direction (0.5 mm). **(D)** Phase diagram of the heart beating for various eGaIn drop volumes on ratchet substrates with three separate directions and different sizes.

Figure 1A shows a schematic representation of the experiment device. The experiment device is composed of six fundamental parts: ratchet substrates, DC voltage, a graphite ring electrode, an inclined plane, an eGaIn drop, and an electrolyte. First, we adjust the angle of inclination to 0.8°, with a glass Petri dish on the middle of the plane. Second, after a graphite ring electrode was placed on ratchet substrates, the whole setup was placed in the middle of the Petri dish. Third, the eGaIn drop was pipetted using a micropipette, the liquid metal lands on the lower side in the graphite ring electrode, and the NaOH solution was poured into the Petri dish until the solution completely covered the surface of the liquid metal drop and graphite ring electrode. Finally, the positive electrode was connected to the graphite ring, and the negative electrode was placed in the electrolyte solution, with the DC power supply voltage (8 V). As shown in Figure 1B, using 3D printing technology, we have successfully prepared a series of ratchet substrates of different sizes. The surface roughness of the sawtooth was about a micrometer scale, as shown in Supplementary Figure S2. To investigate the eGaIn drop heart-beating phenomenon, we set various experimental parameters including the eGaIn volume, ratchet sizes, and directions.

Two typical beating states, regular and irregular beating, are presented in Figure 1C. It shows the frame images of 120 and 60 μL eGaIn drops that are bouncing in the channel direction of the 0.5 mm ratchet structure. The photographs, which display four cycles of continuous beating, are alternatively positioned when the eGaIn drop first contacts the inner side wall of the graphite ring electrode and when the area reaches its maximum after spreading. The colors on the colored marker cards in the figure represent the time required in each of the two consecutive pictures ∆t. When the 120-μL eGaIn drop is beating on the 0.5 mm sawtooth substrate, only one color appears in marker cards, representing the time consumed for each cycle of beating is exactly the same (about 117 ms). As for the images of the beating of the 60-μL eGaIn drop, the marker cards show different colors of changes, indicating changes in the rate of beating.

The eGaIn beating phenomenon varies with the eGaIn drop volume, ratchet size, and movement direction. We set the NaOH concentration (1.5 mol/L), DC voltage value (8 V), inclined plane angle (0.8°), and graphite electrode thickness (1.0 mm), which are constant during the whole experiments. The eGaIn drop volume ranges from 60 to 120 μL. The ratchet size ranges from 0.5 to 2.0 mm. The movement direction includes the leg direction, base direction, and channel direction. As shown in Figure 1D, we depict the eGaIn drop beating phenomenon using a phase diagram. The eGaIn drop beating phenomenon is divided into four regions: region 1 (X) means no beating, region 2 (♦) means regular beating (Supplementary Movie S1), region 3 (▲) means irregular beating (Supplementary Movie S2), and region 4 (●) means beating on the selected direction.

The minimum volume of the eGaIn drop when it is able to beat regularly is 75 μL. When the eGaIn drops are 60 and 65 μL on the ratchet structure larger than 1.0 mm and the eGaIn drop is 70 μL on the 2-mm ratchet structure, the drops are not able to beat. The rest of the less-than 75-μL drops shows irregular beating. When the volume of the eGaIn drop is greater than 70 μL and the ratchet structure is smaller than 2 mm, the drop can beat regularly for all three directions of the ratchet. However, on the 2-mm ratchet substrate, the eGaIn drop can only beat regularly in the partial direction of the substrate, as shown in Supplementary Figure S3.

For the same graphite ring electrode, 20 μL of the eGaIn drop can beat regularly on a smooth surface. The reasons for the difference in the minimum eGaIn drop for regular beating on smooth and ratchet substrates are to consider the interaction between the beating drop and the substrate. First, the small size of the eGaIn drop could fall into the groove of the ratchet structure, which causes a physical disconnection between the drop and the electrode. Second, the eGaIn drop could be blocked by the ratchet structure and does not fall back to the electrode due to the larger friction of the drop on the ratchet than its gravity component. Third, a strong spreading performance of the eGaIn drop is required to initiate the beating due to the ratchet resistance. In contrast to beating on the smooth surface, these factors lead to a larger size of the eGaIn drop on ratchet substrates for regular beating.

### 3.2 Regular and irregular beatings

**FIGURE 2:**
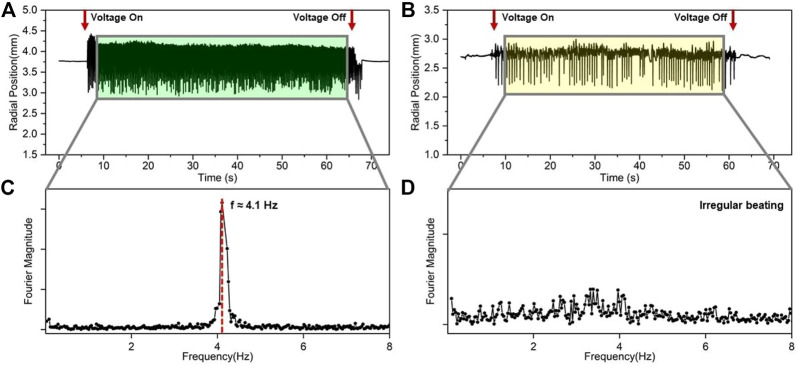
Regular and irregular beatings. **(A)** Schematic diagram of displacement versus time when 120-μL eGaIn drop beatings in the 0.5 mm ratchet substrates in the channel direction. **(B)** Schematic diagram of displacement versus time when 60-μL eGaIn drop beatings in the 0.5 mm ratchet substrates in the channel direction. **(C)** Frequency diagram of the 120-μL eGaIn drop beating in the channel direction on the 0.5 mm ratchet substrate. **(D)** Frequency diagram of the 60-μL eGaIn drop beating in the channel direction on the 0.5 mm ratchet substrate.

By analyzing a large number of frame rate images and using automatic image recognition and tracking in ImageJ, we found the relationship between time and displacement for 120 and 60 μL of the eGaIn drop beating, respectively (Figures 2A, B). These figures show the trajectory of the eGaIn drop when beating in the graphite ring electrode for about 1 minute and record the displacement of the eGaIn drop center relative to the edge of the inner wall of the graphite ring electrode before and after applying the voltage. Without applying the voltage, the eGaIn drop is contacted with the inner side of the electrode, owing to gravity. For the 120-μL eGaIn drop, when voltage is applied, the eGaIn drop starts to move and gradually forms a periodic beating. When the voltage supply is disconnected, the eGaIn drop beats at an irregular frequency within a short period, which is due to the residual charge on the surface of the eGaIn drop. However, for the 60-μL eGaIn drop, there are multiple frequencies of beating. In order to get a clear picture of the beating frequencies of the two drops, the Fourier transform was performed (Figures 2C, D). Figure 2C shows the beating frequency of the 120-μL eGaIn drop is 4.1 Hz, while the beating frequency of the 60-μL eGaIn drop was discrete between 2 and 4 Hz. The reason for the irregular beating is mainly due to the uneven production and consumption of the oxide layer on the surface of the drop at the beginning of the heartbeat.

### 3.3 One heartbeat cycle

**FIGURE 3:**
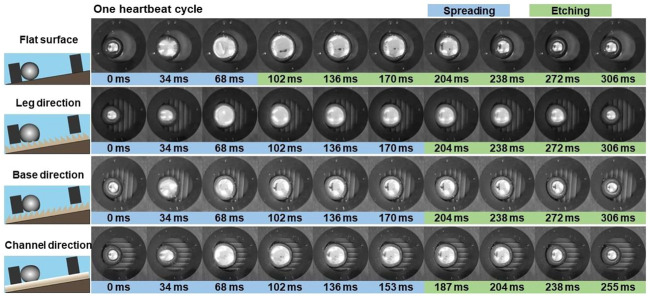
Series of snapshots show the cycle of the 100-μL eGaIn heartbeat on a flat surface and ratchet substrates with three directions, such as leg, base, and channel directions.

To investigate the effect of different substrates, we conducted eGaIn drop heart beating on a flat surface and ratchet substrates in three directions (Figure 3). The ratchet substrates can be divided into three directions, that is, leg, base, and channel directions. In the previous work, the whole beating process was divided into six steps (Yu, Chen, Yun, Cortie, Jiang&Wang 2018a). When DC voltage is turned on, a dynamic interface is formed on the surface of the eGaIn drop due to the simultaneous oxidation and reduction reactions of Ga_2_O_3_ (Formula 1). This leads to a rapid deformation and spreading of the drop as the interfacial tension of the drop decreases sharply. The eGaIn drop separates from the graphite ring electrode after it spreads to its maximum area. The Ga_2_O_3_ layer is rapidly etched in the NaOH solution (Formula 2), which causes switching from a low to high interfacial tension state of the eGaIn drop. Then, the drop becomes a spherical shape and slides to the edge of the electrode to continue the next heartbeat cycle.

$$2Ga+6{OH}^{-}-6e^{-}\;\to \;{Ga}_{2}O_{3}+3H_{2}O,$$ (1)

$${Ga}_{2}\;O_{3}+3H_{2}O+2{OH}^{-}\to 2{\left[Ga{\left(OH\right)}_{4}\;\right]}^{-}.$$ (2)

From the perspective of whether the liquid metal contacts the graphite ring electrode, two steps are defined, which are spreading and etching. The eGaIn drop heart beatings for one cycle on a flat surface, in leg and base directions, are about 306 ms, while it costs 255 ms in the channel direction. The drop can reach the maximum spreading area on the smooth surface (102 ms) faster than those on ratchets (>187 ms). Meanwhile, the maximum spread areas on ratchet substrates are 20% smaller than that on the smooth surface because the resistance from the sawtooth structure hinders the spreading speed and area. In addition, compared with the circular shape of the eGaIn drop on the smooth surface, the shapes of the drops on the ratchet substrates are different when they reach the maximum spreading area. When the eGaIn drop spreads along the channel direction, the drop presents in a square shape. As for the other two directions, the drops present in an elliptical shape.

### 3.4 Oxidative spreading

**FIGURE 4:**
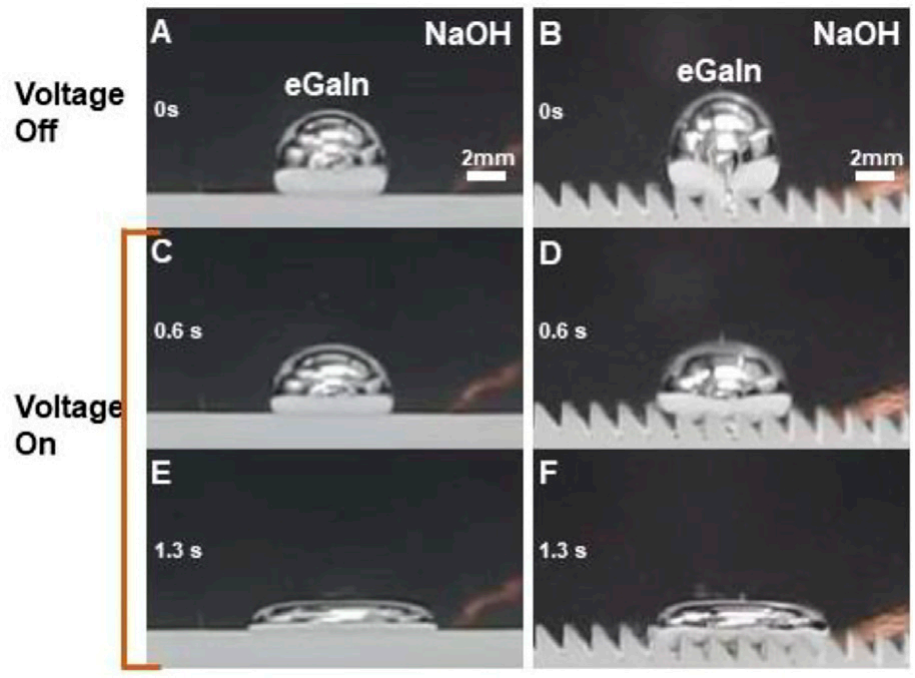
Oxidative spreading of 100-uL eGaIn drops on a smooth surface and a 1.5-mm ratchet substrate in a 1.5 mol/L NaOH solution of 12 V voltage. (A-B) States before power is turned on. (C-D) States after power is turned on for 0.6 s. (E-F) States after power is turned on for 1.3 s.

To evaluate the spreading or wetting behaviors of the eGaIn drop, we conducted it on both smooth and ratchet substrates. Figure 4 shows the oxidative spreading of the 100-μL eGaIn drops on the smooth surface and the 1.5-mm ratchet substrate before and after applying the 12 V DC voltage in a 1.5 mol/L NaOH solution. The eGaIn drops are spherical on both smooth and ratchet substrates, as shown in Figure 4A–F. After applying the voltage, the drops deform suddenly and become flat on the smooth surface, the same as previously reported. For the eGaIn drop on the ratchet substrate, it not only spreads in a long strip but also sinks into the grooves of the sawtooth structure. Obviously, the spread width of the eGaIn drop on the ratchet substrate is narrower than that on the smooth surface.

### 3.5 The eGaIn drop heart beating performance

**FIGURE 5:**
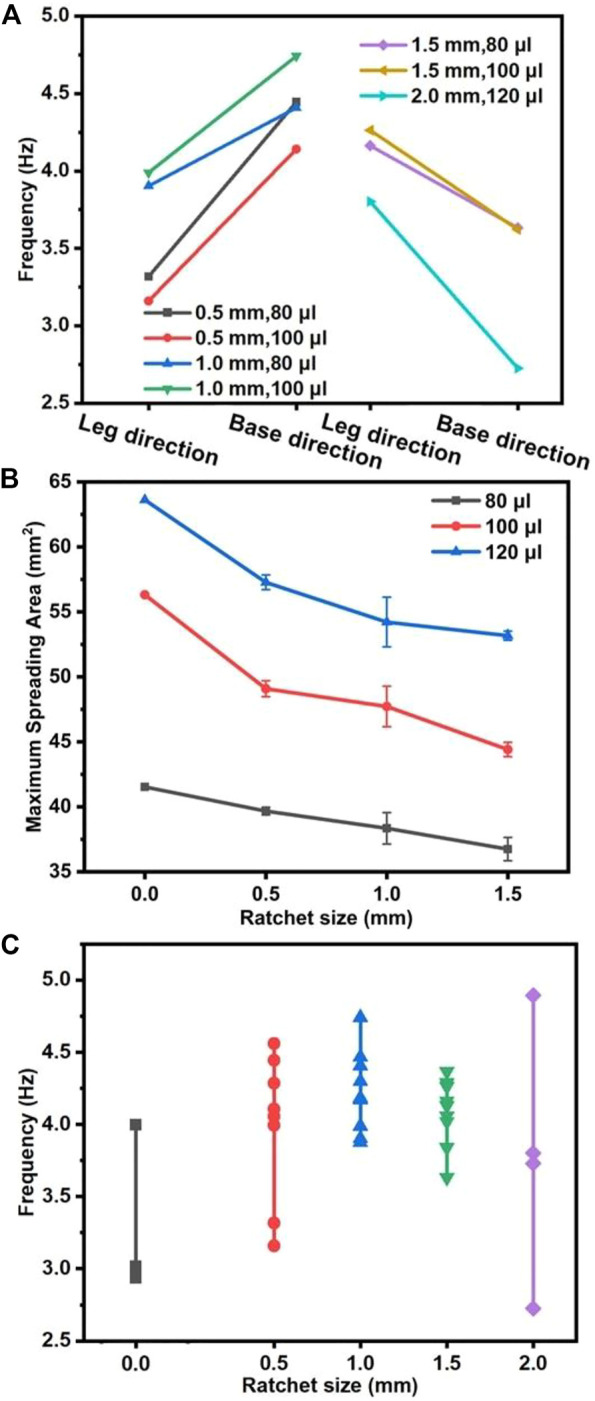
eGaIn drop heart beating performance. **(A)** Beating frequency of eGaIn drops in the base and leg directions. **(B)** Spreading area of the eGaIn drop under different ratchet sizes. **(C)** Beating frequency of the eGaIn drop under different ratchet sizes.

The substrate direction is an important factor that affects the liquid metal heart beating. The experimental results show the beating frequencies fluctuate in a wide range of 2.7–4.7 Hz in the base and leg directions (Figure 5A). When the sawtooth structure is less than 1 mm, the beating frequency for each eGaIn drop in the base direction is larger than that in the leg direction. With the increase in the size of the sawtooth structure, the beating frequency gradually increases. The highest beating frequency is 4.7 Hz when the sawtooth structure is 1.0 mm, and the volume of the drop is 100 μL. However, when the size of the sawtooth structure is larger than 1.5 mm, the trend of the beating frequency is the opposite, whose frequencies in the leg direction are larger than those in the base direction, and the frequency decreases with the increase in the sawtooth structure size. When the sawtooth structure is 2.0 mm, the beating frequency of the eGaIn drop is at least 2.7 Hz. For the beating in the channel direction, the beating frequencies fluctuate around 4.0 Hz.

The ratchet size also has a significant effect on the manipulation of the eGaIn heartbeat frequency and spreading. As the size of the sawtooth structure increases, the maximum spreading area shows a downward trend (Figure 5B). However, with the increase in the eGaIn drop volume from 80 to 120 μL, the maximum spreading area shows an increasing trend because the large size of the sawtooth structure causes a greater resistance to the eGaIn drop spreading. In addition, deeper grooves in the large size of the sawtooth structure allow the eGaIn drop to penetrate into them, resulting in a reduction of the spreading area. On the 1-mm ratchet substrate, the maximum spreading areas of 80, 100, and 120 μL eGaIn drops are about 49, 47.7, and 44.4 mm^2^, respectively. Figure 5C depicts the frequency distribution of the eGaIn drop heart beating on smooth and ratchet substrates. The range of the eGaIn drop beating frequencies on the smooth surface is approximately 3–4 Hz, while the range of the eGaIn drop beating frequency on ratchet substrates is extended. By adjusting the ratchet size, we can selectively control the beating frequency within the range of 2.7–4.8 Hz, which is about 100% larger than that on the smooth surface.

## 4 Conclusion

In conclusion, we designed an asymmetrical sawtooth-structured substrate to improve the manipulation of liquid metal heartbeats with a frequency range that is two times that of the smooth surface, ranging from 2.7 to 4.8 Hz. The images of a single heartbeat cycle show that the maximum spread area of the heartbeat on the sawtooth structure is reduced by 20% compared to the smooth surface. Meanwhile, the spreading speed is also reduced due to the resistance from the sawtooth structure. It was found that eGaIn penetrated into the grooves of the sawtooth structure, and the spread width became narrower than that on the flat surface. The volume of the eGaIn drop that can stably beat on the sawtooth structure is 75 μL. The phase diagram shows the influence of the size of the eGaIn drop and the sawtooth structure on the heartbeat effect, which can be divided into no beating, irregular beating, regular beating, and beating in the selected directions. In addition, the orientation of the sawtooth affects the frequency trend when the sawtooth varies in size. This work enhances our understanding of the manipulation of droplets with high interfacial tension on the ratchet surface. The capability of the liquid metal heartbeat is attractive for a wide range of applications including microfluid pumps, microactuators, switches, and artificial muscle pumps.

## Data Availability

The original contributions presented in the study are included in the article/Supplementary Material; further inquiries can be directed to the corresponding author.
