# Supplementary material for: Electrically driven heartbeat effect of gallium-based liquid metal on a ratchet
Source: Front Bioeng Biotechnol. 2023 Jan 12;10:1094482. doi: 10.3389/fbioe.2022.1094482 (PMC9877452; doi:10.3389/fbioe.2022.1094482)
Supplement: Supplementary file 3 [file DataSheet1.docx]

***Supplementary Material***

**Supplementary Figures**


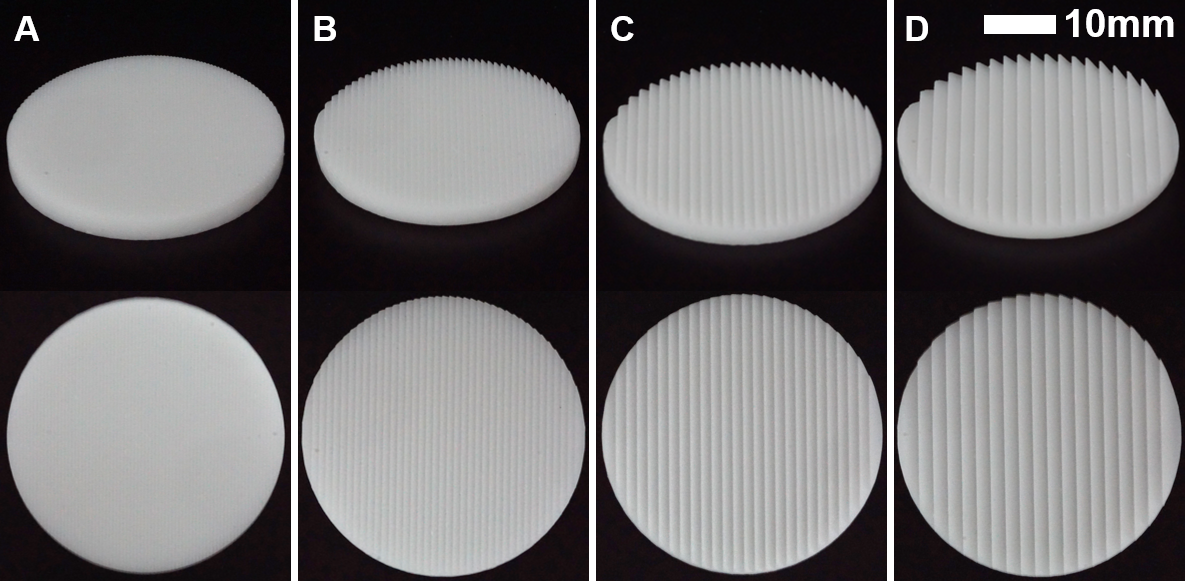


**Supplementary Figure 1.** Optical image of ratchet substrates with the sawtooth sizes from left to right are (A) 0.5 mm, (B)1.0 mm, (C)1.5 mm and (D)2.0 mm.


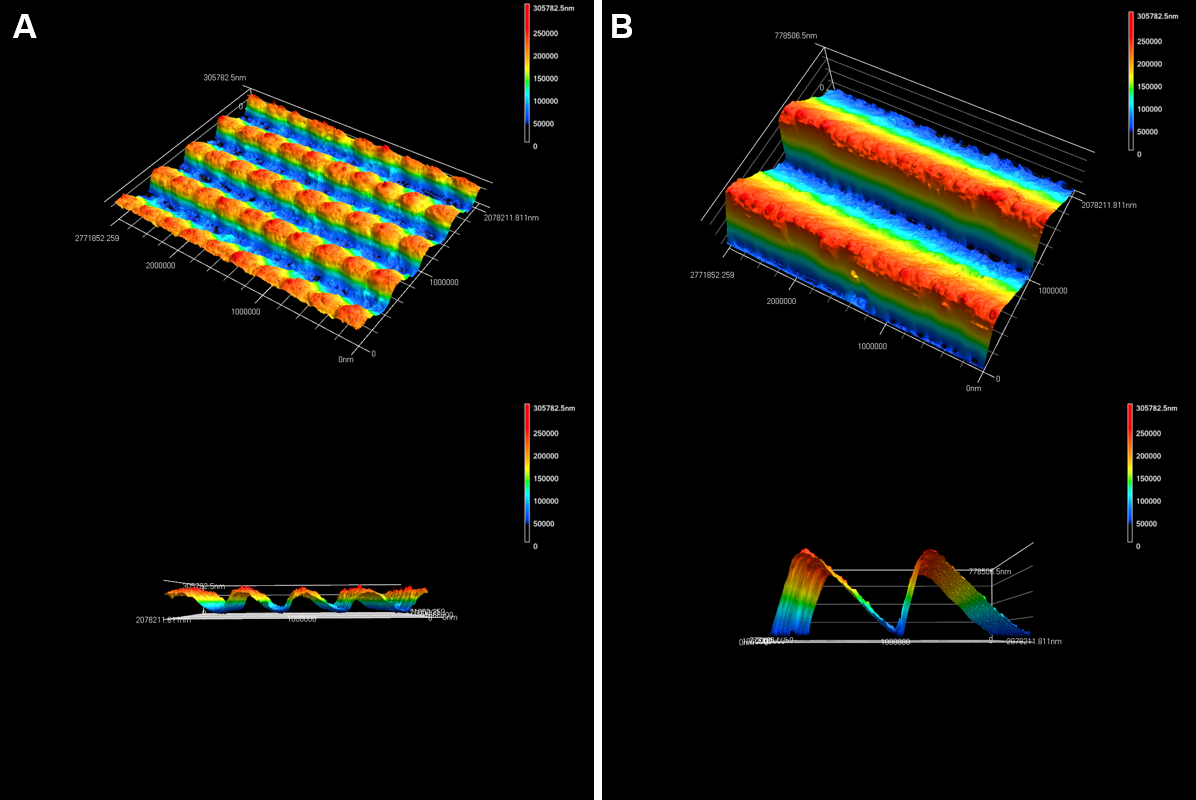


**Supplementary Figure 2.** 3D images and cross-sections of the ratchet substrates with sawtooth sizes of (A) 0.5 and (B)1.0 mm.


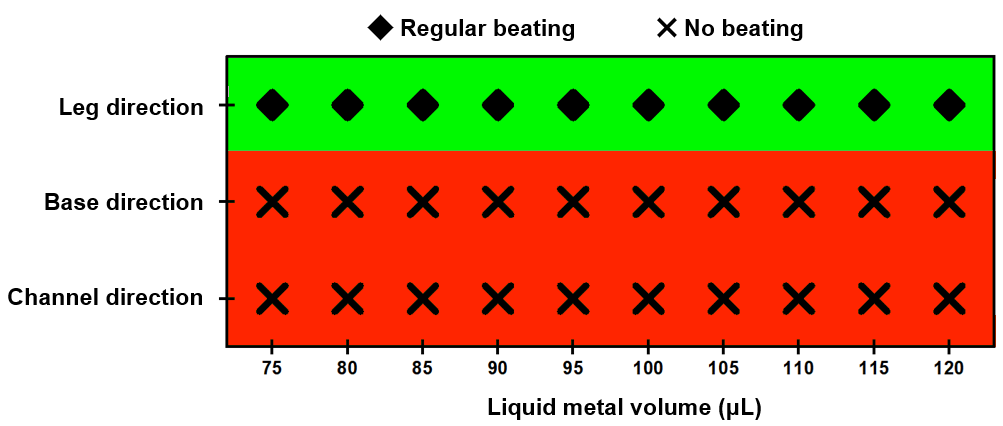


**Supplementary Figure 3.** Phase diagram of the heart beating on 2-mm ratchet substrate with three directions, which is the detail information of Figure 1D region 4.

**Supplementary Movie 1.** Video of 100 μL gallium drop beating regularly in the channel direction of a 0.5 mm ratchet substrate.

**Supplementary Movie 2.** Video of 60 μL gallium drop beating irregularly in the channel direction of a 0.5 mm ratchet substrate.
